# Could self-reported physical performance help predict individuals at the highest risk of mortality and hospital admission events in clinical practice? Findings from the Hertfordshire Cohort Study

**DOI:** 10.1017/S1463423624000173

**Published:** 2024-05-14

**Authors:** Roshan Rambukwella, Leo D. Westbury, Camille Pearse, Kate A. Ward, Cyrus Cooper, Elaine M. Dennison

**Affiliations:** 1 MRC Lifecourse Epidemiology Centre, University of Southampton, Southampton, UK; 2 NIHR Southampton Biomedical Research Centre, University of Southampton and University Hospital Southampton NHS Foundation Trust, Southampton, UK; 3 NIHR Oxford Biomedical Research Centre, University of Oxford, Oxford, UK; 4 Victoria University of Wellington, Wellington, New Zealand

**Keywords:** aging, epidemiology, hospitalization, physical performance

## Abstract

**Aim:**

To consider how self-reported physical function measures relate to adverse clinical outcomes measured over 20 years of follow-up in a community-dwelling cohort (aged 59–73 at baseline) as compared with hand grip strength, a well-validated predictor of adverse events.

**Background:**

Recent evidence has emphasized the significant association of physical activity, physical performance, and muscle strength with hospital admissions in older people. However, physical performance tests require staff availability, training, specialized equipment, and space to perform them, often not feasible or realistic in the context of a busy clinical setting.

**Methods:**

In total, 2997 men and women were analyzed. Baseline predictors were measured grip strength (Jamar dynamometer) and the following self-reported measures: physical activity (Dallosso questionnaire); physical function score (SF-36 Health Survey); and walking speed. Participants were followed up from baseline (1998–2004) until December 2018 using UK Hospital Episode Statistics and mortality data, which report clinical outcomes using ICD-10 coding. Predictors in relation to the risk of mortality and hospital admission events were examined using Cox regression with and without adjustment for sociodemographic and lifestyle characteristics.

**Findings:**

The mean age at baseline was 65.7 and 66.6 years among men and women, respectively. Over follow-up, 36% of men and 26% of women died, while 93% of men and 92% of women were admitted to hospital at least once. Physical activity, grip strength, SF-36 physical function, and walking speed were all strongly associated with adverse health outcomes in both sex- and fully adjusted analyses; poorer values for each of the predictors were related to greater risk of mortality (all-cause, cardiovascular-related) and any, neurological, cardiovascular, respiratory, any fracture, and falls admissions. SF-36 physical function and grip strength were similarly associated with the adverse health outcomes considered.

## Introduction

Aging is associated with a natural decline in physical function. This natural decline is associated with a range of public health issues including an increased level of dependency, premature mortality, risk of falls, and healthcare use (Langhammer *et al.*, 2018). With the demographic shift toward an aging population, there is a growing need to address the health and well-being of older people, particularly their susceptibility to hospital admissions (Simmonds *et al.*, 2014a, Simmonds *et al.*, 2014b). Hospital admissions are not only a significant burden to healthcare systems but also have profound implications for individuals, impacting their quality of life, functional independence, and overall mortality risk. Like many other developed nations, the health system of the UK faces considerable challenges in meeting the needs of older individuals, particularly in the context of hospital admissions.

Recent evidence has emphasized the significant association of physical activity, physical performance, and muscle strength with hospital admissions in older people. Low levels of physical activity in middle age are associated with increased later hospital admission rates, highlighting the potential benefits of regular exercise for people in their midlife (Simmonds *et al.*, 2014a, Luben *et al.*, 2020). Impaired physical performance, such as reduced mobility and balance, has been linked to higher hospital admission risks, suggesting the importance of maintaining functional ability in middle and older ages to prevent health deterioration (Studenski *et al.*, 2003). Moreover, declining muscle strength has been identified as a critical factor contributing to disability among older people, with emerging evidence highlighting the role of higher muscle strength in midlife contributing to overall well-being and disease prevention in later life (Soysal *et al.*, 2021).

However, functional performance and muscle strength tests require staff availability, training, specialized equipment, and space to perform them, often not feasible or realistic in the context of a busy clinical setting. For example, grip strength assessment requires staff training in the procedure, a calibrated dynamometer, a specific chair, and the time to explain the procedure to a patient who is required to complete the task 3 times. Realistically this takes around 10 minutes. If a simpler, quicker, and more feasible screening tool was available, this might help clinicians target resources to those individuals at the highest risk.

To our knowledge, however, no studies have compared self-reported measures of physical function with objective measures regarding their association with adverse health events over 20 years of follow-up among community-dwelling older people. Therefore, in the current study, we considered how self-reported measures (Dallosso physical activity score, SF-36 physical function score, self-reported walking speed) related to adverse clinical outcomes measured over 20 years of follow-up in a community-dwelling cohort aged 59–73 years at baseline, as compared with hand grip strength, a well-validated predictor of adverse events.

## Methods

### The Hertfordshire Cohort Study

The Hertfordshire Cohort Study (HCS) consists of 2997 women and men born in Hertfordshire from 1931 to 1939 and who still lived there in 1998–2004 when they completed a home interview and clinic visit for a detailed health assessment. The HCS received ethical approval from the Hertfordshire and Bedfordshire Local Research Ethics Committee and all participants provided informed consent for the investigations they underwent in 1998–2004 and for researchers to access their medical records in the future. Further details of HCS have been previously published (Syddall *et al.*, 2005, Syddall *et al.*, 2019).

### Ascertainment of participant information at baseline (1998–2004)

Information on smoking, alcohol consumption, and physical activity (Dallosso questionnaire) was ascertained by a nurse-administered questionnaire (Dallosso *et al.*, 1988). Occupational social class was ascertained from most recent or current full-time occupation for men and among women who never married, and from husband’s occupation for ever-married women. Occupations were then classified according to the 1990 Office of Population Censuses and Surveys (OPCS) Standard Occupational Classification (SOC90) unit group for occupation (Office of Population Censuses and Surveys, 1990). Self-reported physical function was assessed according to 10 questions from the physical functioning scale of the SF-36 Health Survey (Ware *et al.*, 1993). Participants reported whether the following 10 activities were limited a little, limited a lot, or not limited at all due to their health: vigorous activities; moderate activities; lifting/carrying groceries; climbing several flights of stairs; climbing one flight of stairs; bending/kneeling; walking at least one mile; walking at least half a mile; walking 100 yards; and bathing/dressing. The total physical function score could range from 0 to 100 with higher scores indicating higher self-reported physical function. Self-reported walking speed was ascertained by asking participants which of the following responses best describes their walking speed: ‘unable to walk’; ‘very slow’; ‘stroll at an easy pace’; ‘normal speed’; ‘fairly brisk’; or ‘fast’.

At the baseline clinic, measurements were made of height (Harpenden pocket stadiometer, Chasmors Ltd, London, UK) and weight (SECA floor scale, Chasmors Ltd, London, UK) and these were used to derive body mass index (BMI). Grip strength was measured using a Jamar dynamometer according to a standardized protocol (Roberts *et al.*, 2011). In brief, the participant was seated with their forearms resting on the chair arms and their wrist just over the end of the chair arm, in a neutral position with the thumbs facing upwards. They were asked to squeeze as hard as they could for as long as they could until the researcher told them to stop. Three trials on each hand were performed with alternating sides and the maximum absolute grip strength value from all six trials was used for analysis.

### Ascertainment of adverse health-related events

Adverse health events were identified using mortality and Hospital Episode Statistics (HES) data. Permission to obtain a HES extract for HCS participants from 01/04/1998 to 31/12/2018 was granted by the Ethics and Confidentiality Committee of the National Information Governance Board and NHS Digital. Linkage of the HCS cohort with HES data has been previously described (Simmonds *et al.*, 2014b); the HES extract included admission information such as the admission date, diagnoses coded to ICD-10, and date of discharge. Adverse health outcomes relating to admissions and deaths that occurred from the HCS baseline clinic (1998–2004) until 31st December 2018 were identified using the ICD-10 codes as stated in Supplementary Table 1.

### Statistical methods

Baseline participant characteristics and the proportion of participants who experienced various adverse health events during follow-up were described using summary statistics. Measures of physical activity, strength, and performance (Dallosso physical activity score, grip strength, SF-36 physical function score, and self-reported walking speed) were examined in relation to risk of adverse health outcomes using time-to-first event Cox regression. Sex-adjusted models and fully adjusted models, accounting for sex, age, height, BMI, smoking status (ever versus never), alcohol consumption, and occupational social class, were implemented; models for grip strength, SF-36 physical function, and self-reported walking speed were also adjusted for physical activity. Adverse health outcomes considered included those specified in Supplementary Table 1. For sensitivity analyses, competing risk analyses for the hospital-related events were performed using the Fine-Gray subdistribution hazards model with death as a competing event (Fine and Gray, 1999).

Associations explored using Cox regression and competing risk analysis were examined among the pooled sample of men and women with adjustment for sex as associations were similar between men and women in sex-stratified analysis (data not shown). Standard deviation scores (*z*-scores) were derived for physical activity, grip strength, and SF-36 physical function and used in analyses. Analyses were performed using Stata, release 17.0; *P* < 0.05 was regarded as statistically significant.

## Results

### Descriptive statistics

Summary statistics for the participant characteristics at baseline and adverse health events during follow-up are presented in Table 1. Mean age at baseline was 65.7 and 66.6 years among men and women, respectively. Mean physical activity scores were similar among men (60.9) and women (59.0), whereas mean grip strength was considerably higher among men (44.0 kg versus 26.5 kg). Median SF-36 physical function scores were higher among men (90) than women (85). Most participants (71% of men and 73% of women) rated their walking speed as normal or higher. During follow-up, 36% of men and 26% of women died, while 93% of men and 92% of women experienced at least one hospital admission.

**Table 1.** Baseline participant characteristics and adverse health events during follow-up

Participant characteristic	Men (*n* = 1579)	Women (*n* = 1418)
***Characteristics at baseline (1998–2004)***		
Age (years)^a^	65.7 (2.9)	66.6 (2.7)
Height (cm)^a^	174.2 (6.5)	160.8 (5.9)
Weight (kg)^a^	82.4 (12.7)	71.4 (13.4)
BMI (kg/m^2^)^a^	27.2 (3.8)	27.6 (4.9)
Ever smoked regularly^c^	67%	39%
Alcohol consumption (units per week)^c^^,^^d^		
Non-drinker	6%	20%
Very low (<1 M & F)	11%	25%
Low (1–10M, 1–7F)	40%	39%
Moderate (11–21M, 8–14F)	21%	11%
Fairly high (22–35M, 15–21F)	11%	3%
High (>35M > 21F)	10%	2%
Dallosso physical activity score^a^	60.9 (15.3)	59.0 (15.7)
Social class (manual)^c^	59%	58%
Grip strength (kg)^a^	44.0 (7.5)	26.5 (5.8)
SF-36 physical function score^b^	90.0 (80.0, 95.0)	85.0 (65.0, 95.0)
Self-reported walking speed^c^		
Fast	4%	6%
Fairly brisk	27%	22%
Normal speed	40%	45%
Stroll at easy pace	24%	20%
Very slow/unable to walk	5%	7%
***Events during follow-up (ever had)^c^***		
Death	36% (34%, 39%)	26% (23%, 28%)
Hospital admission	93% (91%, 94%)	92% (91%, 93%)
***Types of admission during follow-up (ever had)^c^***		
Neurological	23% (21%, 25%)	20% (18%, 22%)
Cardiovascular	71% (68%, 73%)	68% (66%, 71%)
Myocardial infarction	8% (6%, 9%)	5% (4%, 6%)
Stroke	7% (5%, 8%)	5% (4%, 7%)
Respiratory	40% (38%, 43%)	34% (32%, 37%)
Any fracture	9% (8%, 11%)	22% (20%, 24%)
Hip fracture	2% (2%, 3%)	5% (4%, 7%)
Fall	13% (12%, 15%)	21% (19%, 23%)

Follow-up period lasted from baseline (1998–2004) until 31st December 2018.

a Mean (standard deviation).

b Median (lower quartile, upper quartile).

c Percentage (95% confidence intervals are stated for the incident adverse health outcomes).

d M: Male, F: Female.

### Physical activity, strength, and function in relation to risk of adverse health outcomes

Associations between physical activity, grip strength, SF-36 physical function, and self-reported walking speed in relation to adverse health outcomes are presented in Table 2. All four predictors were strongly associated with many of the adverse health outcomes. In both sex- and fully adjusted analyses, poorer values for each of the predictors were related to greater risk of mortality (all-cause, cardiovascular, and other) and the following types of admission: any, neurological, cardiovascular, respiratory, any fracture, and falls. For example, fully adjusted hazard ratios for cardiovascular admissions per standard deviation reduction in physical activity, grip strength, and SF-36 physical function were 1.10 (1.05, 1.15), 1.19 (1.10, 1.29) and 1.31 (1.26, 1.37), respectively; the corresponding hazard ratio per lower band of self-reported walking speed was 1.18 (1.12, 1.24).

**Table 2.** Hazard ratios (95% CI) for physical activity, strength, and function measures in relation to adverse health outcomes

Health outcome	Model	Dallosso physical activity (per lower SD)		Grip strength (per lower SD)		SF-36 physical function (per lower SD)		Self-reported walking speed (per lower band)	
		Hazard ratio (95% CI)	*P*-value	Hazard ratio (95% CI)	*P*-value	Hazard ratio (95% CI)	*P*-value	Hazard ratio (95% CI)	*P*-value
Death (all cause)	Sex-adjusted	**1.29 (1.21, 1.37)**	**<0.001**	**1.53 (1.38, 1.70)**	**<0.001**	**1.41 (1.33, 1.48)**	**<0.001**	**1.44 (1.34, 1.54)**	**<0.001**
	Fully adjusted	**1.23 (1.15, 1.31)**	**<0.001**	**1.39 (1.24, 1.56)**	**<0.001**	**1.28 (1.20, 1.37)**	**<0.001**	**1.31 (1.21, 1.41)**	**<0.001**
Death (cancer)	Sex-adjusted	1.10 (1.00, 1.22)	0.062	**1.20 (1.02, 1.42)**	**0.026**	**1.22 (1.11, 1.34)**	**<0.001**	**1.30 (1.17, 1.45)**	**<0.001**
	Fully adjusted	1.07 (0.96, 1.19)	0.214	1.17 (0.98, 1.40)	0.089	**1.18 (1.06, 1.31)**	**0.002**	**1.29 (1.15, 1.45)**	**<0.001**
Death (cardiovascular)	Sex-adjusted	**1.36 (1.20, 1.54)**	**<0.001**	**1.79 (1.47, 2.19)**	**<0.001**	**1.52 (1.37, 1.69)**	**<0.001**	**1.48 (1.29, 1.69)**	**<0.001**
	Fully adjusted	**1.29 (1.13, 1.46)**	**<0.001**	**1.54 (1.24, 1.92)**	**<0.001**	**1.36 (1.20, 1.53)**	**<0.001**	**1.28 (1.10, 1.49)**	**0.001**
Death (other)	Sex-adjusted	**1.49 (1.33, 1.66)**	**<0.001**	**1.79 (1.49, 2.14)**	**<0.001**	**1.55 (1.42, 1.69)**	**<0.001**	**1.58 (1.40, 1.78)**	**<0.001**
	Fully adjusted	**1.41 (1.26, 1.57)**	**<0.001**	**1.55 (1.28, 1.89)**	**<0.001**	**1.35 (1.21, 1.50)**	**<0.001**	**1.33 (1.17, 1.52)**	**<0.001**
Any admission	Sex-adjusted	**1.13 (1.08, 1.17)**	**<0.001**	**1.21 (1.14, 1.29)**	**<0.001**	**1.34 (1.30, 1.39)**	**<0.001**	**1.21 (1.16, 1.26)**	**<0.001**
	Fully adjusted	**1.11 (1.07, 1.15)**	**<0.001**	**1.21 (1.12, 1.29)**	**<0.001**	**1.31 (1.26, 1.36)**	**<0.001**	**1.16 (1.11, 1.21)**	**<0.001**
Neurological	Sex-adjusted	**1.20 (1.11, 1.30)**	**<0.001**	**1.32 (1.16, 1.50)**	**<0.001**	**1.39 (1.30, 1.49)**	**<0.001**	**1.28 (1.18, 1.39)**	**<0.001**
	Fully adjusted	**1.17 (1.08, 1.27)**	**<0.001**	**1.22 (1.06, 1.40)**	**0.006**	**1.31 (1.21, 1.42)**	**<0.001**	**1.17 (1.07, 1.29)**	**0.001**
Cardiovascular	Sex-adjusted	**1.13 (1.08, 1.18)**	**<0.001**	**1.25 (1.16, 1.34)**	**<0.001**	**1.39 (1.34, 1.45)**	**<0.001**	**1.29 (1.23, 1.35)**	**<0.001**
	Fully adjusted	**1.10 (1.05, 1.15)**	**<0.001**	**1.19 (1.10, 1.29)**	**<0.001**	**1.31 (1.26, 1.37)**	**<0.001**	**1.18 (1.12, 1.24)**	**<0.001**
MI	Sex-adjusted	1.13 (0.98, 1.31)	0.089	**1.55 (1.23, 1.95)**	**<0.001**	**1.30 (1.14, 1.48)**	**<0.001**	**1.36 (1.17, 1.59)**	**<0.001**
	Fully adjusted	1.10 (0.95, 1.28)	0.184	**1.40 (1.09, 1.80)**	**0.008**	**1.22 (1.05, 1.42)**	**0.008**	**1.28 (1.08, 1.51)**	**0.004**
Stroke	Sex-adjusted	**1.19 (1.03, 1.38)**	**0.020**	**1.29 (1.02, 1.64)**	**0.034**	**1.22 (1.06, 1.40)**	**0.006**	0.98 (0.84, 1.15)	0.835
	Fully adjusted	1.15 (0.99, 1.34)	0.068	1.04 (0.80, 1.35)	0.770	1.12 (0.95, 1.31)	0.177	0.88 (0.74, 1.04)	0.142
Respiratory	Sex-adjusted	**1.23 (1.16, 1.30)**	**<0.001**	**1.36 (1.23, 1.50)**	**<0.001**	**1.44 (1.37, 1.51)**	**<0.001**	**1.31 (1.23, 1.40)**	**<0.001**
	Fully adjusted	**1.18 (1.11, 1.26)**	**<0.001**	**1.23 (1.11, 1.37)**	**<0.001**	**1.35 (1.27, 1.43)**	**<0.001**	**1.19 (1.11, 1.27)**	**<0.001**
Any fracture	Sex-adjusted	**1.18 (1.08, 1.30)**	**<0.001**	**1.66 (1.42, 1.95)**	**<0.001**	**1.22 (1.12, 1.33)**	**<0.001**	**1.21 (1.10, 1.34)**	**<0.001**
	Fully adjusted	**1.18 (1.07, 1.30)**	**0.001**	**1.62 (1.37, 1.93)**	**<0.001**	**1.18 (1.07, 1.31)**	**0.001**	**1.20 (1.07, 1.33)**	**0.001**
Hip fracture	Sex-adjusted	1.12 (0.93, 1.35)	0.241	**1.63 (1.19, 2.23)**	**0.002**	1.07 (0.89, 1.29)	0.456	1.22 (1.00, 1.48)	0.051
	Fully adjusted	1.13 (0.93, 1.37)	0.224	**1.68 (1.19, 2.37)**	**0.003**	1.08 (0.87, 1.33)	0.491	**1.28 (1.03, 1.59)**	**0.024**
Fall	Sex-adjusted	**1.20 (1.10, 1.31)**	**<0.001**	**1.64 (1.42, 1.90)**	**<0.001**	**1.32 (1.22, 1.43)**	**<0.001**	**1.36 (1.24, 1.49)**	**<0.001**
	Fully adjusted	**1.16 (1.06, 1.27)**	**0.001**	**1.55 (1.32, 1.81)**	**<0.001**	**1.24 (1.14, 1.36)**	**<0.001**	**1.29 (1.16, 1.43)**	**<0.001**

Health outcomes not referring to deaths all correspond to hospital admission events.

Time-to-first event Cox regression was used; death was regarded as a censoring event for hospital admission outcomes.

Fully adjusted models accounted for sex, age, height, BMI, smoking status (ever versus never), alcohol consumption, and occupational social class; models for grip strength, SF-36 physical function, and self-reported walking speed were also adjusted for physical activity.

Other causes of death were those that were not from cancer or cardiovascular causes.

Standard deviation scores (*z*-scores) were derived for physical activity, grip strength, and SF-36 physical function; estimates are shown per SD reduction in these predictors.

Statistically significant associations (*P* < 0.05) are highlighted in bold.

### Comparison between SF-36 physical function and grip strength regarding associations with adverse health outcomes

These two predictors were similarly associated with the adverse health outcomes considered, with both predictors being significantly associated with 10 out of the 13 outcomes considered in fully adjusted analyses. Hazard ratios were larger for grip strength for most mortality outcomes (all-cause, cardiovascular and other) and for outcomes reflecting poor musculoskeletal health such as any fracture [1.62 (1.37, 1.93) versus 1.18 (1.07, 1.31)], hip fracture [1.68 (1.19, 2.37) versus 1.08 (0.87, 1.33)], and falls [1.55 (1.32, 1.81) versus 1.24 (1.14, 1.36)]. Hazard ratios were larger for SF-36 physical function regarding most other types of hospital admission: any [1.31 (1.26, 1.36) versus 1.21 (1.12, 1.29)]; neurological [1.31 (1.21, 1.42) versus 1.22 (1.06, 1.40)]; cardiovascular [1.31 (1.26, 1.37) versus 1.19 (1.10, 1.29)]; and respiratory [1.35 (1.27, 1.43) versus 1.23 (1.11, 1.37)].

### Sensitivity analyses

Results from competing risk analyses are presented in Supplementary Table 2. Some associations were attenuated compared to when death was treated as a censoring event in time-to-first event Cox regression models. However, all four predictors were related to greater risk of the following types of admission in both Cox regression analyses (Table 2) and competing risk analyses (Supplementary Table 2): any, cardiovascular, respiratory, and falls. This was the case in both sex- and fully adjusted analyses.

## Discussion

This study has demonstrated associations between self-reported measures of physical function when measured at a single time point among older people and adverse outcomes over 20 years of follow-up. For self-reported physical function as a domain of SF-36, associations were of a similar strength compared to using grip strength as a predictor; for example, both lower grip strength and poorer SF-36 physical function were associated with increased risk of mortality (all-cause, cardiovascular, and other) and the any, neurological, cardiovascular, respiratory, any fracture, and falls-related admissions. This is a significant finding, given that grip strength is a well-validated predictor of long-term clinical sequelae (Cheung *et al.*, 2013, Kim *et al.*, 2019).

We did consider other associates of adverse clinical outcomes. For example, the Dallosso physical activity score, as a measure of physical activity levels, demonstrated a strong relationship with the adverse health outcomes considered. Lower scores were consistently associated with an increased risk of hospitalization. This aligns with previous research emphasizing the positive impact of regular physical activity on health outcomes, including the prevention of chronic conditions, improvement in cardiovascular health, and maintenance of functional independence (Simmonds *et al.*, 2014a, Syddall *et al.*, 2016). Our findings emphasize the need to promote and support physical activity programs tailored to older individuals to reduce the likelihood of hospital admissions.

Similarly, self-reported walking speed demonstrated an association with hospital admissions among older people. Slower walking speed was consistently correlated with a higher likelihood of hospitalization. Slower walking speed reflects reduced mobility, impaired physical function, and an increased vulnerability to falls and other adverse health events and can be self-reported or objectively recorded. A systematic review conducted using data from nine longitudinal cohorts in community-dwelling older populations showed that higher measured gait speed of older adults was associated with significant 5-year and 10-year survival, reinforcing its importance (Studenski *et al.*, 2011). In a previous analysis comprising Hertfordshire Cohort Study participants, the relationship between self-reported and measured walking speed and their associations with clinical characteristics and mortality was examined (Syddall *et al.*, 2015). Self-reported walking speed was strongly associated with measured walking speed among men and women (*P* < 0.001) and both measures were similarly associated with clinical characteristics and mortality. This suggests that self-reported walking speed could serve as a useful marker of physical performance in settings where direct measurement of walking speed is not feasible.

Overall, however, the SF-36 physical function score was more consistently associated with adverse health outcomes than physical activity and performed similarly to grip strength regarding its strength of association with these outcomes. As a self-reported questionnaire, the SF-36 Physical Function Survey allows for a relatively quick and efficient assessment of physical function without the need for specialized equipment or extensive training. Furthermore, the SF-36 Physical Function Survey captures various aspects of physical function, including limitations in activities of daily living, mobility, and overall functional independence. This comprehensive assessment provides a holistic understanding of an individual’s physical capabilities and limitations. By identifying areas of impairment, healthcare professionals can address specific functional deficits and develop targeted interventions to mitigate the risk of hospital admissions.

It may be argued that the measurement of grip strength is worthwhile even though the SF-36 physical function score performed similarly to grip strength regarding its strength of association with adverse health outcomes. For example, grip strength is an objective measure and is important for the identification of sarcopenia according to the 2019 European Working Group on Sarcopenia in Older People (Cruz-Jentoft *et al.*, 2019) and the Sarcopenia Definitions and Outcomes Consortium (Bhasin *et al.*, 2020). Low grip strength is associated with increased risk of a variety of adverse health outcomes including physical disability, chronic conditions, and mortality (McGrath *et al.*, 2018). Therefore, we do not propose that grip strength should not be performed under any circumstances; it could definitely be argued that capturing information on SF-36 physical function, grip strength, and Dallosso physical activity during a short clinician-administered session is a justifiable use of resources. However, this may only be possible in some settings, unlike the use of SF-36 physical function scores which have advantages regarding their simplicity, cost-effectiveness, and ability to capture data on a large scale through self-administered questionnaires.

Collecting data on self-reported measures of physical function and adverse health outcomes, such as mortality and types of hospital admission events, in different areas and over time may offer several benefits. For example, comparing how these physical function measures or incident health outcomes differ between regions could identify regions with poorer health that may benefit most from additional resources and interventions, such as targeted health promotion campaigns. Furthermore, data on incident adverse health outcomes could be used to assess the effectiveness of interventions on community health by comparing these incident outcomes before and after the introduction of a new initiative. Finally, longitudinal measurements of an individual’s self-reported physical function may enable early detection of declining health and enable timely health interventions.

This study has several strengths. Our mortality and HES data were comprehensive and not affected by attrition whereby the least healthy members of the cohort are more likely to drop out of the study, as is a common problem in many cohort studies (Howe *et al.*, 2013). However, this study does have some limitations. For example, inpatient admissions were included but outpatient care was not. Furthermore, participants were all Caucasian and from the relatively affluent Southeast of England so findings may be less generalizable to participants of greater socioeconomic disadvantage and those of other ethnicities. However, the demographic, social, and medical characteristics among these participants were similar to those in the nationally representative Health Survey for England (Syddall *et al.*, 2005).

In conclusion, these findings highlight the potential utility of SF-36, a simple screening questionnaire, to identify older adults at the highest risk of adverse clinical outcomes. If administered in the context of a health check, it might be used to identify those who would benefit most from targeted interventions to reduce risk. However, replication of these findings and validation of this tool regarding its predictive capacity is of course required before the introduction of the SF-36 Physical Function Survey into clinical practice.

## Supporting information

Rambukwella et al. supplementary material Rambukwella et al. supplementary material

## Supplementary material

For supplementary material accompanying this paper visit https://doi.org/10.1017/S1463423624000173
