# Supplementary material for: Could self-reported physical performance help predict individuals at the highest risk of mortality and hospital admission events in clinical practice? Findings from the Hertfordshire Cohort Study
Source: Prim Health Care Res Dev. 2024 May 14;25:e25. doi: 10.1017/S1463423624000173 (PMC11091834; doi:10.1017/S1463423624000173)
Supplement: Rambukwella et al. supplementary material [file S1463423624000173sup001.docx]

| **Supplementary Table 1: ICD-10 codes used to define mortality and hospital admission events** | |
| --- | --- |
| **Event** | **ICD-10 codes** |
|  |  |
| ***Mortality events*** |  |
| All-cause mortality | N/A |
| Cancer-related mortality | C00-C97 (death with cancer as the primary underlying cause) |
| Cardiovascular-related mortality | I10-I79 (death with cardiovascular complications as the primary underlying cause) |
| Other mortality events | ICD codes excluding C00-C97 and I10-I79 (death where cancer and cardiovascular complications were not the underlying cause) |
|  |  |
|  |  |
| ***Hospital admission events*** | |
| **Neurological** | G00-G99: Diseases of the nervous system |
| **Cardiovascular** | I10-I79 |
| **Myocardial infarction** | I21: Acute myocardial infarction  I22: Subsequent myocardial infarction |
| **Stroke** | I60: Subarachnoid haemorrhage  I61: Intracerebral haemorrhage  I63: Cerebral infarction  I64: Stroke, not specified as haemorrhage or infarction |
| **Respiratory** | J00-J99: Diseases of the respiratory system |
| **Any fracture** | M80: Osteoporosis with pathological fracture  M84: Disorders of continuity of bone  S22: Fracture of rib(s), sternum and thoracic spine  S32: Fracture of lumbar spine and pelvis  S42: Fracture of shoulder and upper arm  S52: Fracture of forearm  S62: Fracture at wrist and hand level  S72: Fracture of femur  S82: Fracture of lower leg, including ankle  S92: Fracture of foot, except ankle  T02: Fractures involving multiple body regions  T08: Fracture of spine, level unspecified  T10: Fracture of upper limb, level unspecified  T12: Fracture of lower limb, level unspecified  M81: Osteoporosis without pathological fracture  M82: Osteoporosis in diseases classified elsewhere  M83: Adult osteomalacia  M90.7: Fracture of bone in neoplastic disease  S02: Fracture of skull and facial bones  S12: Fracture of neck  T90.2: Sequelae of fracture of skull and facial bones  T91.1: Sequelae of fracture of spine  T91.2: Sequelae of other fracture of thorax and pelvis  T92.1: Sequelae of fracture of arm |
| **Hip fracture** | S72.0 Fracture of neck of femur; Fracture of hip NOS  S72.1 Pertrochanteric fracture; intertrochanteric fracture; trochanteric fracture  S72.2 Subtrochanteric fracture |
| **Fall** | W00-W19: Falls |

| **Supplementary Table 2: Subhazard ratios (95% CI) for physical activity, strength and function measures in relation to adverse health outcomes with death as a competing event** | | | | | | | | | |
| --- | --- | --- | --- | --- | --- | --- | --- | --- | --- |
|  |  | | | | | | |  |  |
| **Hospital admission event** | **Model** | **Dallosso physical**  **activity (per lower SD)** | | **Grip strength**  **(per lower SD)** | | **SF-36 physical**  **function (per lower SD)** | | **Self-reported walking speed**  **(per lower band)** | |
|  |  | **Subhazard ratio (95% CI)** | **P-value** | **Subhazard ratio (95% CI)** | **P-value** | **Subhazard ratio (95% CI)** | **P-value** | **Subhazard ratio (95% CI)** | **P-value** |
| Any admission | Sex-adjusted | **1.12 (1.08,1.17)** | **<0.001** | **1.19 (1.12,1.27)** | **<0.001** | **1.32 (1.26,1.38)** | **<0.001** | **1.18 (1.13,1.24)** | **<0.001** |
|  | Fully-adjusted | **1.10 (1.06,1.15)** | **<0.001** | **1.20 (1.11,1.28)** | **<0.001** | **1.29 (1.23,1.35)** | **<0.001** | **1.14 (1.08,1.19)** | **<0.001** |
| Neurological | Sex-adjusted | **1.15 (1.06,1.24)** | **0.001** | **1.21 (1.06,1.38)** | **0.004** | **1.27 (1.18,1.36)** | **<0.001** | **1.19 (1.09,1.30)** | **<0.001** |
|  | Fully-adjusted | **1.12 (1.03,1.22)** | **0.006** | 1.12 (0.97,1.29) | 0.114 | **1.21 (1.12,1.31)** | **<0.001** | **1.10 (1.00,1.21)** | **0.042** |
| Cardiovascular | Sex-adjusted | **1.10 (1.05,1.15)** | **<0.001** | **1.21 (1.12,1.30)** | **<0.001** | **1.33 (1.27,1.39)** | **<0.001** | **1.24 (1.18,1.30)** | **<0.001** |
|  | Fully-adjusted | **1.08 (1.03,1.13)** | **0.001** | **1.17 (1.08,1.27)** | **<0.001** | **1.26 (1.20,1.33)** | **<0.001** | **1.13 (1.07,1.19)** | **<0.001** |
| MI | Sex-adjusted | 1.09 (0.94,1.26) | 0.239 | **1.44 (1.13,1.83)** | **0.003** | **1.22 (1.08,1.37)** | **0.001** | **1.28 (1.11,1.49)** | **0.001** |
|  | Fully-adjusted | 1.06 (0.92,1.23) | 0.414 | **1.32 (1.02,1.71)** | **0.036** | **1.16 (1.00,1.33)** | **0.043** | **1.21 (1.03,1.43)** | **0.019** |
| Stroke | Sex-adjusted | 1.13 (0.98,1.31) | 0.095 | 1.18 (0.93,1.50) | 0.161 | 1.12 (0.97,1.28) | 0.118 | 0.92 (0.78,1.10) | 0.357 |
|  | Fully-adjusted | 1.10 (0.94,1.27) | 0.228 | 0.96 (0.73,1.25) | 0.745 | 1.03 (0.88,1.21) | 0.713 | 0.83 (0.69,1.01) | 0.061 |
| Respiratory | Sex-adjusted | **1.20 (1.13,1.27)** | **<0.001** | **1.29 (1.17,1.42)** | **<0.001** | **1.37 (1.30,1.44)** | **<0.001** | **1.25 (1.18,1.34)** | **<0.001** |
|  | Fully-adjusted | **1.15 (1.08,1.23)** | **<0.001** | **1.16 (1.05,1.29)** | **0.005** | **1.29 (1.21,1.37)** | **<0.001** | **1.13 (1.05,1.22)** | **0.001** |
| Any fracture | Sex-adjusted | **1.12 (1.02,1.24)** | **0.015** | **1.52 (1.29,1.79)** | **<0.001** | **1.13 (1.03,1.23)** | **0.006** | **1.13 (1.03,1.25)** | **0.013** |
|  | Fully-adjusted | **1.13 (1.02,1.24)** | **0.015** | **1.50 (1.26,1.79)** | **<0.001** | **1.10 (1.00,1.21)** | **0.043** | **1.12 (1.01,1.24)** | **0.026** |
| Hip fracture | Sex-adjusted | 1.05 (0.87,1.27) | 0.623 | **1.44 (1.10,1.89)** | **0.007** | 0.98 (0.81,1.18) | 0.823 | 1.12 (0.92,1.35) | 0.266 |
|  | Fully-adjusted | 1.07 (0.87,1.30) | 0.526 | **1.51 (1.10,2.06)** | **0.010** | 0.98 (0.80,1.20) | 0.857 | 1.18 (0.97,1.44) | 0.090 |
| Fall | Sex-adjusted | **1.13 (1.04,1.24)** | **0.006** | **1.50 (1.30,1.73)** | **<0.001** | **1.21 (1.12,1.30)** | **<0.001** | **1.26 (1.14,1.38)** | **<0.001** |
|  | Fully-adjusted | **1.11 (1.01,1.21)** | **0.028** | **1.42 (1.21,1.66)** | **<0.001** | **1.15 (1.05,1.26)** | **0.002** | **1.20 (1.08,1.33)** | **0.001** |
|  |  |  |  |  |  |  |  |  |  |
| A competing risk analysis was implemented using the Fine-Gray subdistribution hazards model; death was regarded as a competing event | | | | | | | | |  |
| Fully-adjusted models accounted for sex, age, height, BMI, smoking status (ever vs never), alcohol consumption and occupational social class; models for grip strength, SF-36 physical function and self-reported walking speed were also adjusted for physical activity | | | | | | | | | |
| Subhazard ratios of greater than one indicate that poorer values were related to greater incidence of the adverse event; subhazard ratios of less than one correspond to reduced incidence | | | | | | | | | |
| Standard deviation scores (z-scores) were derived for physical activity, grip strength and SF-36 physical function; estimates are shown per SD reduction in these predictors  Statistically significant associations (p<0.05) are highlighted in bold | | | | | | | | |  |
